# Application of Nerve Stimulation: Current Status and Future Directions

**DOI:** 10.3390/brainsci15050475

**Published:** 2025-04-29

**Authors:** Hee Young Kim, Younbyoung Chae

**Affiliations:** 1 Department of Physiology, Yonsei University College of Medicine, Seoul 03722, Republic of Korea; 2 Acupuncture and Meridian Science Research Center, Kyung Hee University, Seoul 02447, Republic of Korea

Neuromodulation is frequently used to modulate neuronal activity and influence brain function. However, to advance precision treatment methods, it is crucial to understand the interaction between nerves and organs. Acupuncture can stimulate peripheral nervous systems that correspond with neuromodulation techniques [1,2]. There is considerable overlap in both the practice and theoretical foundations of acupuncture and neuromodulation [3].

Thirteen papers in this Special Issue address the present status and future direction of various nerve stimulation applications. Two review articles highlight key aspects: one summarizes the role of the hypothalamus in acupuncture’s effects on pain, stress, and metabolic regulation (Contribution 1), while the other discusses the cognitive and affective aspects of acupuncture needling from the standpoint of the Bayesian brain (Contribution 2). Both reviews shed light on how acupuncture exerts multifaceted therapeutic effects across a range of diseases.

Three papers on animals explore potential mechanisms by which acupuncture or electro-acupuncture can reduce pain and aid recovery from ischemic stroke. The application of lifting and thrusting needle manipulation at ST36 increased the mechanical pain threshold in an inflammatory pain rat model through p38 MAPK-mediated F-actin (Contribution 3). By inactivating TRPV1 and IL-17-related pathways, electro-acupuncture at ST36 decreased pain-like behaviors in a fibromyalgia animal model (Contribution 4). Acupuncture at GV26 and PC6 alleviated infarction and neurological deficits by preventing blood–brain barrier disruption and regulating autophagy–apoptosis (Contribution 5). These investigations contribute to our understanding of acupuncture’s mechanisms and the development of potential therapeutic strategies of neuromodulation.

Numerous neuromodulation techniques have been successfully used in randomized clinical studies to treat patients with a range of illnesses. Repetitive transcranial magnetic stimulation (rTMS) applied to the parietal cortex enhanced sleep quality and reduced periodic leg movements in patients with Parkinson’s disease (Contribution 6). The mechanical stimulation of the triceps surae muscle complex modulated muscular hypertonia, which is crucial for rehabilitation treatment (Contribution 7).

Two studies focus on the objective measurement of treatment progress in patients and the challenges of accurately diagnosing visceral functions. Increased variability in cutaneous reflex amplitudes is associated with symptoms of chronic ankle instability (Contribution 8). Significant differences in muscle tone, stiffness, and pain sensitivity were observed across five back-shu points, which are believed to be clinically important for the diagnosis of illnesses of visceral organs (Contribution 9).

Numerous non-invasive neuromodulation methods have been applied in diverse clinical contexts, including transcutaneous auricular vagus nerve stimulation (taVNS), transcranial direct current stimulation (tDCS), and transcutaneous electrical spinal cord stimulation (tSCS) (Contributions 10–12). Interestingly, two distinct neuromodulation modalities—tDCS and acupuncture—were applied to patients with chronic low back pain, revealing both shared and distinct patterns of cerebral blood flow changes in brain networks involved in pain processing and modulation (Contribution 13).

We believe that this Special Issue of *Brain Sciences*, which delves into the neural mechanisms underlying various neuromodulation tools and their diverse applications in patients with various disorders, will be enjoyable to readers. Future studies should focus on establishing the optimal dosage for each neuromodulation tool for personalized medicine and maximizing their effects by leveraging the synergistic potential of several neuromodulation modalities [4]. These findings, in our opinion, could contribute to the connection between various forms of nerve stimulation and both fundamental and applied research.
